# Criterion and Convergent Validity of Youth Physical Activity and Sedentary Behavior Questionnaires in School Settings: A Systematic Review of Current Evidence and Future Perspectives

**DOI:** 10.3390/children13070931

**Published:** 2026-07-15

**Authors:** Mégane Gagnon, Salma Bouqartacha, Éloane Bédard, Livia Delattre, Martin Descarreaux, Roseane de Fátima Guimarães

**Affiliations:** 1 Department of Human Kinetics, Université du Québec à Trois-Rivières, 3351 des Forges Blvd. P.O. Box 500, Trois-Rivières, QC G8Z 4M3, Canada; megane.gagnon@uqtr.ca (M.G.); eloane.bedard@uqtr.ca (É.B.); livia.delattre@uqtr.ca (L.D.); martin.descarreaux@uqtr.ca (M.D.); 2 Department of Anatomy, Université du Québec à Trois-Rivières, 3351 des Forges Blvd. P.O. Box 500, Trois-Rivières, QC G8Z 4M3, Canada; salma.bouqartacha@uqtr.ca

**Keywords:** physical activity, sedentary behavior, questionnaire validity, children and adolescents, systematic review

## Abstract

**Highlights:**

**What are the main findings?**

• This systematic review identified 22 studies evaluating 16 questionnaires used to measure physical activity and sedentary behavior among children and adolescents in school settings, with validity estimates ranging from very weak to strong.
• Eight questionnaires demonstrated the most acceptable evidence for criterion validity, convergent validity, and reliability.

**What are the implications of the main findings?**

• Researchers and practitioners should use caution when selecting physical activity and sedentary behavior questionnaires, as many commonly used instruments lack sufficient evidence of measurement accuracy and may not accurately reflect true behavior levels.
• Further high-quality validation studies are needed to strengthen the evidence base and improve confidence in questionnaire-based assessments of physical activity and sedentary behavior in children and adolescents.

**Abstract:**

Background/Objectives: Physical activity and sedentary behavior are key indicators of youth health, and questionnaires offer a cost-effective method for data collection. This systematic review synthesized evidence on the criterion and convergent validity of self-report questionnaires measuring physical activity and sedentary behavior in children and adolescents aged 5–18 years in school settings. Methods: Searches were conducted in SPORTDiscus, Medline, Scopus, ERIC, Academic Search Complete, Education Source, and the Cochrane Library with no date limits. Included studies compared questionnaire results with established measures and were assessed using the consensus-based standards for the selection of health measurement instruments (COSMIN) checklist. Results: Twenty-two studies covering 16 questionnaires were included; additional outcomes included test–retest reliability, content and construct validity, and psychometric evaluation. Validity correlations ranged from very weak to strong (r = 0.11–0.80; ρ = 0.20–0.87), with Bland–Altman agreement (ρ = 0.56–0.70) and mean absolute percentage error up to 199.6%. Conclusions: Eight questionnaires showed acceptable validity and reliability, though research is needed to strengthen evidence on measurement accuracy.

## 1. Introduction

It is well-established that physical activity (PA), and sedentary behavior (SB) are critical determinants of children’s and adolescents’ physical and mental health [1]. PA, which is any bodily movement produced by skeletal muscles requiring energy expenditure, increases physical fitness and reduces the risk of obesity, all while providing meaningful benefits to psychological well-being and cognitive performance [1,2,3,4]. On the other hand, SB, especially when combined with increased screen time, is associated with weaker psychosocial and academic profiles [4,5]. Thus, reduced PA and increased SB can have detrimental effects on public health.

The Canadian 24 h movement guidelines for children and youth recommends at least 60 min per day of moderate-to-vigorous PA, involving a variety of aerobic activities incorporated with vigorous activity and muscle and bone strengthening exercises at least three times a week [5]. Also, the guidelines recommend limiting leisure screen time to a maximum of two hours per day and reducing prolonged periods of sedentary behavior [5]. Adherence to these guidelines is typically monitored in school settings using proxy measures, such as self-reports [6], and less frequently through objective measures, such as accelerometry [7].

Evaluating youth activity profiles directly within school settings provides valuable insight into adherence to these guidelines in a context where children spend a substantial portion of their daily time. Moreover, it allows for assessment guidelines to be implemented in schools and can inform future interventions and public policy. When assessing PA and SB in daily life, movement sensors offer a precise and objective means of capturing the duration, frequency, and intensity of movement [8]. However, to gain insight into the specific types and contexts of PA and SB, self-reports are often used [9,10]. Each method has distinct strengths and limitations. For example, pedometers and accelerometers may not accurately capture certain types of activity, such as cycling, aquatic activities, or static exercises (e.g., upper-body movements), whereas self-report questionnaires are susceptible to recall bias and may be influenced by social desirability [8]. Questionnaires can be used in large populations at a low cost [11]; however, given the frequent discrepancies observed between objective measures and self-reported data, there is a clear need for accurate, context-sensitive tools to strengthen behavioral surveillance efforts. As questionnaires remain among the most widely used instruments for assessing PA and SB, it is essential to evaluate their quality. A notable gap in recent research is the limited assessment of these tools with regard to their criterion and convergent validity [9,10].

Validity refers to the extent to which a measure accurately reflects the construct it is intended to assess [12]. In this context, criterion validity describes how well a proxy measurement tool corresponds to a well-established and reliable reference standard (criterion) [12]. Establishing criterion validity is particularly important, as it supports the use of more accessible and cost-effective instruments to approximate constructs that would otherwise require more complex or resource-intensive methods [12,13]. Convergent validity refers to the extent to which a measurement tool is associated with another measure intended to assess the same construct, although neither is considered a definitive criterion [14]. In the context of PA and SB assessment (in or out of the school setting), objective measurement tools commonly used for such comparisons include heart rate monitors, accelerometers, and pedometers [10].

Validating PA and SB questionnaires in children and adolescents can be challenging because of age-related cognitive limitations in their ability to recall their own behavior, as well as in their interpretation of the correct PA intensity [11]. Additionally, setting-specific factors such as school schedule structure and screen use patterns, combined with the typically unstructured nature of PA among children and adolescents, may influence their understanding and limit the ability of questionnaires to accurately assess their activity profiles [11].

Previous reviews have evaluated the validity of PA and SB questionnaires in a variety of settings among youth populations albeit separately by groups of age [10,15,16]. Many of the studies do not distinguish between questionnaires used in general contexts and those specifically targeting the school setting, where many of children’s and adolescents’ daily PA and SB occur [9,10]. Moreover, the methodologies used across validation studies vary considerably, limiting direct comparisons and conclusions regarding instrument reliability. Finally, the evaluation of criterion and convergent validity is underrepresented in current research [9,16].

Given these limitations, there is a clear need for a focused synthesis of the criterion and convergent validity of PA and SB questionnaires designed for use in school environments. Such a review is particularly relevant for researchers seeking to select or develop accurate measurement tools, educators implementing activity monitoring or school-based interventions, and policymakers requiring reliable data to inform health promotion strategies. By focusing on instruments validated within school settings and across different school-age groups, this review will enhance their applicability in these contexts and support the effective monitoring of health-related lifestyle behaviors.

The aim of this study is to systematically evaluate the criterion and convergent validity of questionnaires measuring PA and SB in children and adolescents within school environments.

## 2. Materials and Methods

This systematic review is reported in accordance with the Preferred Reporting Items for Systematic Reviews and Meta-Analyses (PRISMA) criteria [17]. The protocol for this systematic review was registered at the International Prospective Register for Systematic Reviews (PROSPERO) with the registration number CRD420251125556.

### 2.1. Eligibility Criteria

To be included in the review, articles had to meet the following criteria: (1) feature questionnaires (self-reported); (2) comprise school-aged children and adolescents (5–18 years old); (3) evaluate criterion or convergent validity (using an objective measure, such as accelerometers, pedometers, doubly labeled water [DLW], and direct observation); (4) were in English; and (5) should provide information on both measurements (PA and SB). Studies were excluded if they met any of the following criteria: (1) systematic reviews/metanalysis, case reports, comments, or editorials; (2) interviews or diaries; (3) animal research; (4) no criterion or construct measure; and (5) papers published in languages other than English.

### 2.2. Search Strategy

Systematic literature searches were carried out in 7 major electronic databases (Medline, SPORTDiscus, Scopus, ERIC, Academic Search Complete, Education Source, and Cochrane Library). Searches were conducted between July and August 2025. The research question for this review was defined using Population, Exposure, Comparison and Outcome (PECO) criteria (Table 1): among school-aged children and adolescents (5–18 years), how valid are self- or proxy-reported questionnaires (paper or electronic) for assessing PA and SB in a school setting when evaluated against objective and convergent measures?

Search terms were used in AND combinations, which related to questionnaires (questionnaire* OR instrument* OR measure* OR survey* OR scale OR checklist* OR form* OR assessment OR tool* OR self-reported OR self-administered OR proxy OR recall OR test), physical activity (activit* OR “physical activit*” OR exercis* OR “motor activity” OR sport* OR leisure OR “active play”), sedentary behavior (sedentar* OR “sedentary behavio*” OR inactivit* OR “sedentary lifestyle” OR sitting OR screen-time), school setting (school* OR classroom* OR “middle school” OR “high school” OR “elementary school” OR educ* OR “educational institution” OR academy), children and adolescents (child* OR adolescen* OR youth OR kid OR kids OR teen* OR scholar* OR schooler* OR boy* OR girl*), and criterion and convergent (“objective measure” OR pedometer OR acceleromet* OR “sense wear” OR “gold standard” OR “doubly labeled water” OR DLW OR “direct observation” OR monitor* OR validity OR feasibility OR reliability).

Searches were adapted to each database, alongside the use of appropriate Boolean operators and database-specific filters (see Supplementary Materials S1). References and citation searches of included studies, as well as checking reference lists of the selected existing reviews, were conducted for completeness.

### 2.3. Selection Procedures

Titles and abstracts were screened for eligible studies by two independent researchers (M.G.) and (S.B.). Subsequently, full texts were then obtained and screened for eligibility by M.G. and S.B. A third researcher (R.d.F.G.) was consulted in cases of doubt.

### 2.4. Data Extraction and Management

The bibliographic files were imported into the Covidence systematic review software (2026), Veritas Health Innovation (Melbourne, Australia), to facilitate the screening process. The first step was to remove duplicates. Following this, the initialed researchers assessed the titles and abstracts of the remaining studies. Based on our predefined eligibility criteria, studies were either rejected for not meeting the criteria or accepted for a more in-depth review of their methodology sections. Following the selection of the final studies, two independent researchers (M.G. and S.B.) extracted data regarding the characteristics of studies and results of the assessed measurement properties using a structured form.

Data extracted from the studies included the authors, year of publication, country of the sample, sample size and age range, the questionnaire used, the comparison measure, the statistical analysis performed, and the main results for physical activity and sedentary behavior. In cases of disagreement regarding data extraction, a third researcher (R.d.F.G.) was consulted.

### 2.5. Methodological Quality Assessment

Two independent researchers (L.D. and R.d.F.G.) rated the methodological quality of the included studies using the standardized consensus-based standards for the selection of health measurement instruments (COSMIN) checklist [18] (see Supplementary Materials S2). For each measurement property, the design requirements were rated using a 4-point scale (i.e., excellent, good, fair, or poor). The lowest score counts method was applied, e.g., the final methodological quality was scored as poor in the case of a poor score on one of the items. The lowest rated items that determined the final score for each study are shown in Supplementary Materials S2. One minor adaption to the original COSMIN checklist, also described in a previous review [9], was applied; Percentage of Agreement (PoA) was removed from the reliability box and added to the measurement error box as an excellent statistical method [19].

## 3. Results

**Figure 1:**
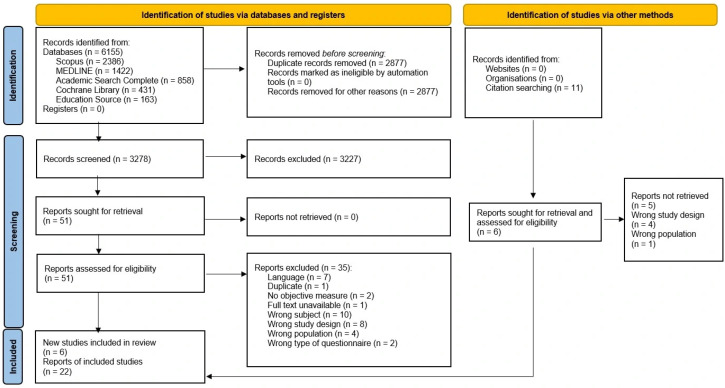
PRISMA 2020 flow diagram of study selection.

The initial search identified 6155 studies from the databases investigated. After excluding duplicates (n = 2877), 3278 titles and abstracts were screened, and 3227 titles and abstracts from published studies were excluded based on the inclusion criteria. Then, 51 studies were screened in full, and 35 were excluded. Finally, 16 studies were eligible for inclusion after screening was completed. After reviewing all reference lists, six additional studies were retrieved, resulting in a total of 22 papers included in this systematic review (Figure 1).

Within the 22 studies, five assessed construct validity [20,21,22,23,24], 11 assessed criterion validity [20,23,25,26,27,28,29,30,31,32,33], two assessed concurrent validity [34,35], two assessed convergent validity [36,37], two assessed cross-validation [38,39], 15 assessed test–retest reliability [20,21,22,23,24,25,26,27,28,29,31,32,34,36,37], and one assessed measurement error [38]. In these studies, 16 included different questionnaire assessments. The YAP questionnaire was assessed in three studies; the DABQ was assessed in one study; the CAPAS questionnaire was assessed in one study; the IPAQ was assessed in three studies; the PASBAQ was assessed in one study; the MARCA questionnaire was assessed in two studies; the PAQ-C questionnaire was assessed in one study; the PAQ-A questionnaire was assessed in two studies; the eMLTPAQ questionnaire was assessed in one study; the YPAS questionnaire was assessed in one study; the PASE questionnaire was assessed in one study; the PDPAR-24 questionnaire was assessed in one study; the GEMS questionnaire was assessed in one study; the GAQ was assessed in two studies; the AQuAA questionnaire was assessed in one study; and one study used a “home-made” questionnaire (no official name). Overall, validity correlations ranged from very weak to strong (r = 0.11 to 0.80; ρ = 0.20 to 0.87). Bland–Altman analyses showed moderate agreement, with wide limits of agreement spanning −1404 to 1614 kcal/day and 0.1 to 5.6 MJ·day^−1^. A weighted kappa reported in one study indicated fair agreement (κ = 0.41), while MAPE values reached up to 199.6%, suggesting substantial variability in estimation error across instruments. Details of each study and related questionnaires are presented in Table 2.

Table 3 summarizes the evidence on the validity of self-reported measurement tools compared with objective methods. Overall, self-reported measures often diverge from objective estimates for MVPA, total PA, and SB; however, these discrepancies vary in nature, encompassing both systematic bias (e.g., consistent over- or underestimation) and random error. While some tools may consistently underestimate or overestimate values, indicating stable but biased measurements, others exhibit greater variability.

## 4. Discussion

The aim of this review was to systematically evaluate the criterion and convergent validity of questionnaires measuring PA and SB in children and adolescents within school settings. This systematic review identified 22 studies examining the measurement properties of questionnaires used to assess PA and SB in this population, providing an overview of the questionnaires assessed and summarizing the evidence for their criterion and convergent validity.

There is considerable variation among questionnaires, as different constructs were assessed through the selected studies, with some questionnaires measuring PA in terms of time spent, while others use energy expenditure, step counts, or intensity levels [22,25,29,40,41,42]. Recall periods also vary widely across studies and questionnaires, ranging from 1 [22,25,42] to 28 days [31]. Short recall periods may improve accuracy but capture less typical behavior, while longer periods may better reflect habitual activity but increase memory error. This trade-off can systematically affect reported PA levels and between-study comparability. Differences in recall windows may partially explain inconsistent associations between PA and health outcomes across studies [43]. Also, the timing of when a questionnaire is sent can affect both the number and the quality of responses.

In addition, the format differs substantially across questionnaires, including the number of items, which range from 6 [27] to 63 [41] questions. Short instruments may be more feasible for large-scale surveillance but risk oversimplifying PA behavior. Questionnaires with more questions may capture more dimensions (e.g., domains, intensity, context) but can increase fatigue and potential response error [1]. In addition to questionnaire length, there was heterogeneity in how PA was quantified across tools. For instance, the PAQ-A and PAQ-C (for adolescents and children) rely on a 1 to 5 Likert scale, producing summary scores rather than direct estimates of PA or SB [20]. This limits interpretability and makes comparisons with objective measures more challenging.

Most studies had low risks of bias but limited evidence for criterion and convergent validity. Many studies had positive validity ratings (YAP; YPAS; DBAQ; PAQ-A; eMLTPAQ; “home-made” questionnaire; MARCA; CAPAS-Q) but medium risks of bias (see Supplementary Materials S2). Small sample sizes, instrument calibration, confounding variables, poor question wording, recall bias, and social desirability are all common methodological issues that can influence the quality results of the studies included in this review (see Supplementary Materials S2).

Only seven questionnaires had high methodological quality and positive evidence for criterion and/or convergent validity (YAP; YPAS; DBAQ; PAQ-A; eMLTPAQ; “home-made” questionnaire; MARCA) (see Supplementary Materials S2). Therefore, this observation may reflect limitations in validation processes (e.g., small sample sizes, absence of a gold standard, limited statistical analyses). Some questionnaires may have been widely used despite insufficient validation. This highlights the need to apply more rigorous methodological standards in both the development and validation of such instruments [44].

Questionnaires’ validity was reported with correlations ranging from “very poor” to “very strong”. Only eight valid questionnaires were identified with good evidence regarding PA and SB: YAP; YPAS; DBAQ; PAQ-A; eMLTPAQ; “home-made” questionnaire; MARCA; and CAPAS-Q. This suggests that there is no single superior questionnaire; instead, multiple instruments exist that are suited to different contexts, including format, population, and methodological approach. Despite notable limitations in validation, these questionnaires continue to be widely used to estimate PA and SB in children and adolescents [9,10].

In summary, this review shows that the criterion and convergent validity of questionnaires assessing PA and SB in school-aged populations is highly variable and often limited. Although several instruments demonstrate promising validity, there was a lack of consistency, combined with methodological and conceptual heterogeneity; thus, we have identified eight questionnaires with better evidence for criterion and convergent validity [20,22,25,26,28,30,34,36,38,39,40,41].

### 4.1. Strengths and Limitations

A key strength of this review is that data extraction and quality assessment were conducted independently by multiple researchers, thereby reducing the risk of bias and enhancing the reliability of the findings. Furthermore, cross-referencing of the included studies was performed to maximize the identification and inclusion of the relevant literature. However, limitations should be acknowledged. First, only studies published in English were included, which may have led to the exclusion of relevant research available in other languages. Second, the inclusion criteria required questionnaires to assess both PA and SB. As a result, validated instruments focusing exclusively on either PA or SB may have been overlooked. Finally, it is also important to acknowledge that variability between accelerometry devices, including sensor drift, differences in proprietary algorithms, and variations in sensor placement on the body, may substantially influence the quality and comparability of the data collected, and consequently may impact the outcomes reported across studies.

### 4.2. Recommendations for Future Research

Future research should prioritize rigorous validation protocols and clearer alignment between constructs and measurement methods to improve the reliability and interpretability of self-reported PA and SB data.

To improve future research, we recommend the use of standardized frameworks, such as COSMIN, for the evaluation of measurement properties, as this would enhance the methodological quality and comparability of validation studies. Researchers should consider that the mode of administration used in validation studies should reflect its intended use in real-world settings. It is also essential to clearly define the context of use and the underlying measurement model of a questionnaire to determine which measurement properties are most relevant for assessments. Greater emphasis should be placed on evaluating content validity to ensure that questionnaires are comprehensive and adequately capture the constructs of interest. Instead of developing new instruments, efforts should prioritize the refinement and further validation of the most promising existing questionnaires. In addition, providing open access to the evaluated questionnaires would improve transparency and facilitate their broader use. Finally, we encourage journal editors to require authors and reviewers to adopt standardized tools, such as COSMIN, when conducting and appraising studies on measurement properties.

## 5. Conclusions

We conclude that eight questionnaires were identified with conclusive evidence for acceptable validity or reliability (moderate-to-good results) for both PA and SB. Studies that use PA and SB questionnaires should acknowledge that their findings may not accurately reflect the true PA levels of children and adolescents, as many of these questionnaires lack sufficient evidence of validity. More attention to the criterion and convergent validity of PA and SB questionnaires is needed to confirm that questionnaires measure their intended constructs.

## Figures and Tables

**Table 1:** PECO criteria for inclusion of studies.

Parameter	Criterion and Convergent
Population	School-aged children and adolescents (5–18 years)
Exposure	Self-report or proxy-report questionnaires (paper or electronic)
Comparison	Objective criterion and convergent measures
Outcome	Criterion and convergent validity metrics

**Table 2:** Criterion or convergent validity of self-reported physical activity (PA) and sedentary behavior (SB) for youth, sorted by study sample information, comparison measure, and level of evidence.

Author and Year of Publication	Questionnaire	Study Sample	Objective Measure	Statistical Analysis	PA Results	SB Results
Kastelic et al., 2022. [36]	Daily Activity Behaviors Questionnaire (DABQ)	n = 58 15–18 y.o.	ActivPAL4 (PAL Technologies Ltd., Glasgow, Scotland)	Spearman’s correlation (ρ)	The correlation between MVPA and total physical activity ranged between 0.50 and 0.53 The correlation for LPA was ρ = 0.25	Moderate correlation for SB (ρ = 0.38)
Fillon et al., 2022. [34]	Children and Adolescents Physical Activity and Sedentary Questionnaire (CAPAS-Q)	n = 120 8–18 y.o.	ActiGraph GT3X accelerometer (ActiGraph, LLC, Pensacola, FL, USA)	Spearman’s correlation (ρ)	The correlation between CAPAS-Q and PA is moderate (ρ = 0.45)	The correlation between CAPAS-Q and SB was moderate (ρ = 0.38)
Fairclough et al., 2019. [38]	Youth Activity Profile (YAP)	n = 402 10–16 y.o.	SenseWear Armband Mini (SWA)	Pearson’s correlation (r); mean absolute percentage error (MAPE)	In-school MVPA: weakly correlated (r = 0.11), and the MAPE was 70.8% Out-of-school MVPA: moderately correlated (r = 0.45), and the MAPE was 83.9% Weekend MVPA: moderately correlated (r = 0.52), and the MAPE was 199.6%	Out-of-school SB: strongly correlated to SB estimated from SWA (r = 0.80), and MAPE was 50.6%
Saint-Maurice et al., 2017. [30]	Youth Activity Profile (YAP)	n = 628 12–17 y.o.	ActiGraph GT3X+ accelerometer (ActiGraph, LLC, Pensacola, FL, USA)	Pearson’s correlation (r)	MVPA at school was moderately correlated (r = 0.38) MVPA accumulated during out-of-school time was moderately correlated (r = 0.52) MVPA based on weekend items were not significantly correlated (r = 0.16)	Predicted SB was moderately correlated with GT3X+ data (r = 0.32)
Vandoni et al., 2017. [33]	International Physical Activity Questionnaire (IPAQ)	n = 30 16–20 y.o.	Actiheart monitor (CamNtech Ltd., Cambridge, UK)	Spearman’s correlation (ρ)	VPA showed a strong correlation (ρ = 0.62) For moderate PA, there was no correlation (ρ = 0.23)	Did not find any significant correlation between the two measurements relevant to sedentary behavior (ρ = −0.02)
Aggio et al., 2016. [20]	PAQ-A	n = 169 11–17 y.o.	ActiGraph GT1M accelerometer (ActiGraph, LLC, Pensacola, FL, USA)	Spearman’s correlation (ρ)	Total daily PA showed a moderate correlation (ρ = 0.42) and daily MVPA a weak correlation (ρ = 0.39)	The results include both PA and SB
Saint-Maurice et al., 2015. [39]	YAP	n = 291 8–16 y.o.	SenseWear Armband Pro3 (SWA).	Pearson’s correlation (r)	School estimates were moderately correlated (r = 0.58) Activity scores were not significantly correlated (r = 0.19) Out-of-school activity was also aggregated into weekend activity estimates These two estimates were not significantly correlated (r = 0.22)	Estimates of sedentary time were strongly correlated (r = 0.75)
Scholes et al., 2014. [31]	Physical Activity and Sedentary Behavior Assessment Questionnaire (PASBAQ)	175 16 y.o.	KineSoft software (KineSoft, Saskatoon, SK, Canada)	Spearman’s correlation (ρ)	Total time spent for PA was moderately correlated (ρ = 0.30) in girls and weakly correlated in boys (ρ = 0.20)	Total sedentary time was moderately correlated (girls: ρ = 0.30; boys: ρ = 0.25)
Wang et al., 2013. [37]	IPAQ—Short Form	n = 1021 12–18 y.o.	ActiGraph GT3X+ accelerometer (ActiGraph, LLC, Pensacola, FL, USA)	Spearman’s correlation (ρ)	Weak correlation was found in total PA (ρ = 0.31) and in MVPA (ρ = 0.22)	SB correlation was very weak (ρ = 0.18)
Foley et al., 2013. [22]	Multimedia Activity Recall for Children and Adolescents (MARCA)	n = 32 10–18 y.o.	Total energy expenditure using doubly labeled water	Spearman’s correlation (ρ); Bland–Altman plots (limits of agreement)	A strong correlation was observed for TEE (ρ = 0.70) and a moderate correlation for AEE (ρ = 0.56) MARCA over-estimated TEE by an average of 50 kcal/day (limits of agreement, −1589 to 1490 kcal/day) and underestimated AEE as 105 kcal/day (limits of agreement, −1404 to 1614 kcal/day).	The results of AEE and TEE include both PA and SB
Bringold-Isler et al., 2012. [26]	A “home-made” questionnaire surveying physical activity and sedentary behavior (no official name)	n = 189 6–14 y.o.	ActiGraph AM7164 accelerometer (ActiGraph, LLC, Fort Walton Beach, FL, USA)	Spearman’s correlation (ρ)	PA was moderately correlated (ρ = 0.46)	SB was moderately correlated (ρ = 0.55)
McVeigh et al., 2012. [28]	PAQ-C	n = 30 9–11 y.o.	Actical accelerometer (Philips Respironics, Bend, OR, USA)	Pearson’s correlation (r); weighted kappa (κ) (degree of disagreement)	A moderate correlation was found (r = 0.53) The ability of PAQ to correctly categorize children into activity levels was moderate (κ = 0.41)	A strong correlation was found for time spent doing sedentary activities between both measures (r = 0.63)
Belton et al., 2010. [25]	Youth Physical Activity Self-Report (YPAS)	n = 47 7–9 y.o.	Polar Team System HR monitor; direct observations (DOs) of participants’ physical activities were carried out by trained observers (CPAF observation system)	Spearman’s correlation (ρ)	A strong correlation was found between self-reported activity intensity and HR: ρ = 0.87 for weekday and ρ = 0.795 for weekend Corresponding correlations for activity duration were ρ = 0.837 and ρ = 0.684 for weekday and weekend, respectively	The results of self-reported activity, HR, and DOs include both PA and SB
Chinapaw et al., 2009. [21]	Activity Questionnaire for Adolescents and Adults (AQuAA)	n = 42 12–16 y.o.	MTI ActiGraph accelerometer (Model 7164; ActiGraph LLC, Pensacola, FL, USA)	Spearman’s correlation (ρ)	Negatively associated with moderate-to-vigorous activities (ρ = −0.23)	SB was weakly correlated (ρ = 0.23)
Jago et al., 2009. [27]	Physical Activity Self-Efficacy (PASE)	n = 714 6th grade students (83 with accelerometers)	MTI ActiGraph accelerometer (Model 7164; ActiGraph LLC, Pensacola, FL, USA)	Pearson’s correlation (r)	Full and reduced scales had weakly non-significant correlations with accelerometer counts per minute after school for boys (r = 0.18), with comparable associations for girls (r = 0.16) Non-significant weaker correlations were observed between PASE and minutes of MVPA (r = 0.09–0.11)	Negatively associated with sedentary time (r = −0.29) when using the full set of IRM items
Rangul et al., 2008. [29]	IPAQ	n = 71 13–18 y.o.	ActiReg monitor (PreMed AS, Oslo, Norway) and cardiorespiratory fitness test	Spearman’s correlation (ρ)	For TEE, there was a very weak correlation with vigorous activity (ρ = −0.14) and moderate activity (ρ = 0.01) For VO_2peak_, there was a weak correlation for vigorous activity (ρ = −0.32) and a very weak correlation for moderate activity (ρ = 0.13)	For TEE and VO_2peak_, there was a very weak correlation for sitting (ρ = −0.04; ρ = 0.18)
Trost et al., 2007. [35]	PDPAR-24	n = 122 13.8 +/− 1.2 y-o.	Yamax Digi-Walker electronic pedometers (SW-700 and SW-200; Yamax Corporation, Tokyo, Japan)	Spearman’s correlation (ρ)	Positive correlations were observed between all three PDPAR-24 variables (30 min blocks VPA, 30 min blocks MVPA) and daily step counts (ρ = 0.29 to 0.34)	Inverse correlation was observed between self-reported screen time and daily step counts (ρ = −0.19)
Ridley et al., 2006. [23]	MARCA	n = 1429 9–15 y.o.	ActiGraph accelerometer (Model AM7164-2.2C; ActiGraph LLC, Fort Walton Beach, FL, USA)	Spearman’s correlation (ρ)	There is a weak correlation between MVPA (ρ = 0.35) and locomotion (ρ = 0.37)	There was a moderate correlation for PAL with screen time (ρ = 0.45)
Arvidsson et al., 2005. [40]	Physical Activity Questionnaire for Adolescents (PAQA)	n = 33 15–17 y.o.	Doubly labeled water (DLW) and indirect calorimetry (RMR)	Pearson’s correlation (r); Bland–Altman plots (limits of agreement)	There was a strong correlation (r = 0.62) between EE_PAQA_ and EE_DLW_ PAQA underestimated energy expenditure by 3.8 (1.7) MJ (limits of agreement not specified in text)	Strong correlation between predicted and measured RMR (r = 0.85)
Treuth et al., 2004. [32]	Girls’ Health Enrichment Multi-Site Studies (GEMS) Activity Questionnaire—(GAQ)	n = 172 8–10 y.o.	ActiGraph accelerometer (Model 7164WAM; ActiGraph LLC, Fort Walton Beach, FL, USA)	Pearson’s correlation (r)	A low correlation was found between GAQ’s usual activity scores and average ActiGraph minutes of MVPA, namely between 12 noon and 6 PM for the total sample (r = 0.11) and the comparison group (r = 0.15)	Correlations for SB were not statistically significant
Slinde et al., 2003. [41]	Extended Minnesota Leisure Time Physical Activity Questionnaire (eMLTPAQ)	n = 35 15–17 y.o.	Total energy expenditure using doubly labeled water	Spearman’s correlation (ρ); Bland–Altman plots	A moderate correlation was found between TEE_DLW_ and EE_LTPA_ (ρ = 0.49) eMLTPAQ underestimated TEE with a mean difference of 2.8 MJ·d1 (limits of agreement: 0.1 to 5.6 MJ·d1)	Including questions about inactivity increased the correlation to ρ = 0.65; predicted BMR (indirect calorimetry) was the one that correlated best with eMLTPAQ (r^2^ = 0.73)
Treuth et al., 2003. [24]	GEMS Activity Questionnaire (GAQ)	n = 68 8–9 y.o.	MTI/CSA accelerometer Manufacturing Technology Inc. (formerly Computer Science and Applications, Fort Walton Beach, FL, USA)	Pearson’s correlation (r)	The correlations (all 28 activities) were very weak and non-significant (r = −0.05 to 0.21) A weak correlation was found when reducing to 18 physical activities (r = 0.27 to 0.29)	Correlations between TV watching and sedentary activity (excluding TV) were very weak (r = −0.004 to −0.145; r = −0.09 to 0.02)

Note: PA = physical activity; SB = sedentary behavior; VPA = vigorous physical activity; MVPA = moderate-to-vigorous physical activity; LPA = light physical activity; TEE = total energy expenditure; AEE = activity energy expenditure; PAL = physical activity level; HR = heart rate; DO = direct observation; VO_2peak_ = peak oxygen uptake; RMR = resting metabolic rate; BMR = basal metabolic rate; DLW = doubly labeled water; IRM = item response model; κ = kappa coefficient; r = Pearson’s correlation coefficient; ρ = Spearman’s correlation coefficient; MAPE = mean absolute percentage error; SWA = SenseWear Armband.

**Table 3:** Summary table of validity evidence of the measurement tools.

Method	Outcome Measures					Comparison Results	Authors and Year of Publication
	LPA	MVPA	Total PA	SED	Steps		
ActiGraph (GT3X)	-	-	♀ 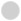 ♂ 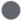	♀ 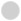 ♂ 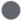	-	Total PA: ∆ min/day (median estimates) ♀ −188.6 (*p* < 0.001) ♂ −178.0 (*p* < 0.001) SED: ∆ minutes/day ♂ −122.1 (*p* < 0.001) ♀ −145.0 (*p* < 0.001)	Scholes et al., 2014 [31]
ActiGraph (GT3X)	-	School: 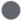	-	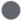	-	MVPA: ∆ min/week –17.8 (*p* = 0.31) SED: ∆ min/week −75.6 (*p* = 0.02)	St-Maurice et al., 2017 [30]
ActiGraph (GT3X)	-	-	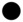	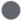	-	Total PA: ∆ min/day ♀ 240 (*p* < 0.001) ♂ 298 SED: ∆ min/day ♀ 551 ♂ 519	Fillion et al., 2022 [34]
ActiGraph (GT3X)	-	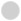	-	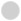	-	Usual PA: Accelerometry counts per minute * Baseline IG: 381 Baseline CG: 354 Usual PA: MET-weighted GAQ * Baseline IG: 3.05 Baseline CG: 2.87	Treuth et al., 2004 [32]
ActiGraph (GT3X)	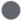	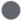	-	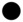	-	MVPA: ∆ min/day ♀ 121.7 (*p* < 0.001) ♂ 152.5 SED: ∆ min/day Screen ♀ 203.8 (*p* < 0.001) ♂ 273.7	Ridley et al., 2006 [23]
ActiGraph (GT3X)	-	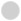	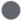	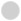	-	Average Min/day MVPA (IPAQ-SF) = 57.19 MVPA (Actigraph) = 29.06 Average Min/day SED (IPA-SF) = 587.52 SED (Actigraph) = 555.96	Wang et al., 2013 [37]
ActiGraph (MTI)	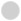	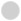	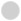	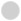		No descriptives reported	Jago et al., 2009 [27]
ActivPAL	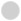	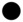	-	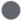	-	Average Min/day MVPA (DABQ2) = 53 MVPA (ActivPAL) = 76 Total PA (DABQ2) = 418 Total PA (ActivPAL) = 271 SED (DABQ2) = 563 SED (ActivPAL) = 719	Kastelic et al., 2022 [36]
Actical	-	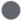	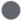	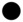	-	Average time of their day MPA (PAQ) = 18% of their day MPA (Actical) = 20% of their day Average Min/day VPA (PAQ) = 13.3 VPA (Actical) = 19.7 Average time of their day SED activity (PAQ) = 58% of their day SED activities (Actical) = 54% of their day	McVeigh and Norris, 2012 [28]
DLW	-	-	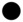	-	-	∆ kcal / day TEE: 50 (*p* < 0.05) AEE: −105 (*p* < 0.05)	Foley et al., 2013 [22]
ActiGraph (GT3X)	-	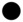	-	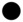	-	Average min/day Total MVPA (Actigraph): 153.4 Median min/day Total MVPA (self-reported): 361.4 Average min/day Total SED (Actigraph): 482.9 Median min/day Total SED (self-reported): 197.1	Bringold-Isler et al., 2012 [26]
SW Armband Mini (SWA)	-	In-school: 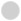 Out-of-school: 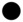 Weekend: 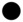	-	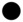	-	∆ min/week (equivalence zone) MVPA in-school: 17.2 (20%) MVPA out-of-school: 31.6 (20%) MVPA weekend: −4.9 (15%) ∆ min/week (equivalence zone) SED out-of-school: 109.2 (15%)	Fairclough et al., 2019 [38]
Actiheart	-	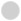	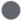	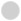	-	∆ min/day Walking + moderate PA: −17.6 (*p* < 0.05) Vigorous PA: −5.1 SED: 209.2 (*p* < 0.05)	Vandoni et al., 2017 [33]
Polar HR monitor	-	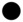	-	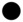	-	No descriptives reported	Belton et al., 2010 [25]
Pedometer (SW-700 & SW-200)	-	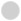	-	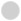	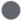	Mean steps * Group 1 = 14,559 Group 2 = 12,116 24h period PA Mean METs * Group 1 = 2.0 Group 2 = 2.1	Trost et al., 2007 [35]
DLW	-	-	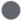	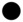	-	∆ MJ·d^−1^ eMLTPAQ underestimated TEE Mean difference of 2.8 MJ·d1	Slinde et al., 2003 [41]
SW Armband Pro3 (SWA)	-	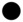	-	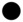	-	∆ min/week MVPA in-school: −15.6 MVPA out-of-school weekdays: 3.4 MVPA out-of-school weekend: −21.7 SED out-of-school on weekdays: −49.7	Saint-Maurice et al., 2015 [39]
ActiGraph (MTI, 7164 model )	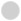	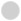	-	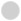	-	AQuAA (median (25th–75th percentile)) Adolescents AQuAA score (MET * min/wk): 8464 (5146;8465) SED activities (min/wk): 3000 (2415;3600) Light activities (min/wk): 810 (600;1335) Moderate activities (min/wk): 565 (348;1019) Vigorous activities (min/wk): 35 (0;155) Accelerometer (median (25th–75th percentile)) Adolescents Counts/min: 430 (339;510) SED activities (min/wk): 4838 (4602;5076) Light activities (min/wk): 910 (764;1165) Moderate activities (min/wk): 43 (11;66) Vigorous activities (min/wk): 1 (0;4)	Chinapaw et al., 2009 [21]
ActiGraph (GT1M model)	-	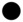	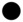	-	-	Modified PAQ-A scores (mean +/− SD) Full sample: 2.8 ± 0.6 Accelerometry median and interquartile range Total daily PA (mins/d) (median and interquartile range): Full sample: 221.6 [193.1–241.6] Daily MVPA (mins/d): Full sample: 57.1 [42.2–71.0] Daily sedentary time (mins/d): Full sample: 484.5 [458.6–515.7]	Aggio et al., 2016 [20]
DLW	-	-	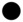	-	-	EE_DLW_ (MJ/day) (median (range)): ♀: 8.7 (7.3–11.9) ♂: 11.1 (9.1–13.9) EE_DLW/BW_ (kJ/day) (median (range)): ♀: 164 (132–208) ♂: 178 (133–225) EE_DLW/FFM_ (kJ/day) (median (range)): ♀: 217 (197–259) ♂: 202 (183–255) EE_PAQA_ (MJ/day) (median (range)): ♀: 4.9 (3.9–8.4) ♂: 7.2 (5.3–11.2)	Arvidsson et al., 2005 [40]
Accelerometer MTI/ CSA	-	-	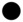	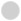	-	MTI/CSA mean (count-min^−1^) (SD): Day1: 410.3(173.7) Day2: 409.2 (152.7) Day3: 365.0 (138.3) Day4 373.8 (113.0) Activitygram mean (intensity-min) (SD): Day1: - Day2: 266.87 (252.0) Day3: 316.8 (279.7) Day4: 216.6 (212.1)	Treuth et al., 2003 [24]
ActiReg + cardiorespiratory fitness test (VO_2peak_)	-	-	VO_2peak_ 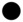 TEE 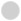 PAL 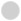	VO_2peak_ 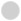 TEE 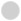 PAL 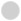	-	Physical fitness test: mean (SD) VO_2peak_ (L·min^−1^): 3.04 (0.77) VO_2peak_ (mL·kg^−1^·min^−1^): 52.54 (8.12) Actireg: mean (SD) Actireg (PAL for 7 days): 1.70 (0.24) Actireg (TEE for 7 days): 59.39 (8.65) ActiReg (min at METs < 3 for 7 days): 8954 (441) ActiReg (min at METs 3–6 for 7 days): 845 (313) ActiReg (min at METs > 6 for 7 days): 256 (210) IPAQ: mean (SD) Vigorous activity (days/week): 2.76 (1.84) Vigorous activity (min/day): 73 (43) Moderate activity (days/week): 2.89 (2.18) Moderate activity (min/day): 65 (42) Walking (days/week): 4.39 (2.19) Walking (min/day): 43 (53) Sitting (min/day): 374 (196)	Rangul et al., 2008 [29]

Correlation values: 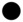 good = ≥0.40; 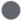 moderate = 0.3–0.39; 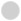 weak = ≤0.29. Kappa values: 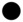 good = ≥0.60; 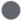 moderate = 0.4–0.6; 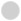 fair/weak = <0.20. * Not comparable units. LPA = light physical activity; MVPA = moderate-to-vigorous physical activity; PA = physical activity; SED = sedentary behavior; ∆ = mean difference; min = minutes; wk = week; d = day; MET = metabolic equivalent of task; kcal = kilocalories; MJ = megajoules; TEE = total energy expenditure; AEE = activity energy expenditure; EE = energy expenditure; PAL = physical activity level; HR = heart rate; DLW = doubly labeled water; VO_2peak_ = peak oxygen uptake; BW = body weight; FFM = fat-free mass; IG = intervention group; CG = control group; SD = standard deviation; SWA = SenseWear Armband.

## Data Availability

The data are available upon reasonable request from the authors.

## Supplementary Materials

The following supporting information can be downloaded at: https://www.mdpi.com/article/10.3390/children13070931/s1, Table S1: Database searches; Table S2: Quality assessment.

## Abbreviations

The following abbreviations are used in this manuscript:

PA	Physical activity
SB	Sedentary behavior
