# Supplementary material for: Criterion and Convergent Validity of Youth Physical Activity and Sedentary Behavior Questionnaires in School Settings: A Systematic Review of Current Evidence and Future Perspectives
Source: Children (Basel). 2026 Jul 15;13(7):931. doi: 10.3390/children13070931 (PMC13406170; doi:10.3390/children13070931)
Supplement: Supplementary file 1 [file children-13-00931-s001.zip › Table S1 Database searches.pdf]

Supplementary material s1: Database searches, Boolean operators and database specific filters

(questionnaire* OR instrument* OR measure* OR survey* OR scale OR checklist* OR form* OR assessment OR tool* OR self-reported OR self-administered OR proxy OR recall OR test) AND (activit* OR "physical activit*" OR exercis* OR "motor activity" OR sport* OR leisure OR "active play" in Title Abstract Keyword AND school* OR classroom* OR "middle school" OR "high school" OR "elementary school" OR educ* OR "educational institution" OR academy) AND (child* OR adolescen* OR youth OR kid OR kids OR teen* OR scholar* OR schooler* OR boy* OR girl*) AND ("objective measure" OR pedometer OR acceleromet* OR "sense wear" OR "gold standard" OR "doubly labeled water" OR DLW OR "direct observation" OR monitor* OR validity OR feasibility OR reliability)

Table S1. Thesaurus/Mesh terms for each database :

Database	Vocabulary Type	Physical Activity / Sport	Sedentary Behavior	Population	School Context	Measurement Tools	Validity / Reliability	Number of Articles
SPORTDiscus / EBSCOhost	Descriptors (DE)	DE “School recess breaks” OR DE “School sports” OR DE “School exercises & recreations” OR DE “After school sports” OR DE “Physical activity” OR DE “Exercise”	DE “Sedentary behavior in children” OR DE “Sedentary lifestyles” OR DE “Sedentary behavior”	DE “Children” OR DE “Youth” OR DE “High school students” OR DE “Students” OR DE “School children”	DE “Education”	DE “Accelerometers” OR DE “Pedometers”	-	431
MEDLINE / EBSCO	MeSH (MH)	MH “Sports” OR MH “Youth Sports” OR MH “Exercise”	MH “Sedentary Behavior”	MH “Child” OR MH “Adolescent”	MH “Schools” OR MH “Education”	MH “Surveys and Questionnaires”OR MH “Form”	MH “Reproducibility of Results”	1421
Scopus / Elsevier	TITLE-ABS-KEY (free search)	TITLE-ABS-KEY (“physical activity” OR “exercise” OR “school sport*“)	TITLE-ABS-KEY (“sedentary behavior”	TITLE-ABS-KEY (“child” *OR*	TITLE-ABS-KEY (“school*” OR “education”)	Included in free search	TITLE-ABS-KEY (“validity” OR “reliability”)	2386

			OR “sedentary lifestyle”)	*”adolescent*” OR “youth”)				
ERIC / EBSCO	Descriptors (DE)	DE “Physical Activities” OR DE “Recreational Activities” OR DE “Playground Activities” OR DE “Physical Activity Level” OR DE “Extracurricular Activities” OR DE “School Activities”	No specific term used	DE “Elementary School Students” OR DE “Middle School Students” OR DE “High School Students” OR DE “Secondary School Students” OR DE “Students” OR DE “Youth” OR DE “Adolescents” OR DE “Young Adults”	Included via school population	DE “Questionnaires” OR DE “Surveys” OR DE “School Surveys” OR DE “Student Surveys” OR DE “Self Evaluation (Individuals)”	DE “Validity” OR DE “Test Validity” OR DE “Reliability” OR DE “Test Reliability” OR DE “Criterion Referenced Tests” OR DE “Criterion Validity (Predictive)” OR DE “Standardized Tests”	116
Academic Search Complete / EBSCO	Descriptors (DE)	DE “Sports” OR DE “School sports” OR DE “After school sports” OR DE “Sports for children” OR DE “Physical activity” OR DE “Exercise”	DE “Sedentary behavior in children” OR DE “Sedentary lifestyles” OR DE “Sedentary behavior”	DE “Children” OR DE “Youth”	DE “Middle school education” OR DE “High school students” OR DE “Elementary schools” OR DE “Primary schools” OR DE “Education”	DE “Accelerometers” OR DE “Pedometers” OR DE “Youth Self-Report”	DE “Validity” OR DE “Test validity”	858

Education Source / EBSCO	Descriptors (DE)	DE “After school sports” OR DE “School sports” OR DE “Exercise” OR DE “Exercise for children”	-	DE “Children” OR DE “Youth”	DE “School sports” OR DE “Schools”	DE “Youth Self-Report” OR DE “Student surveys”	DE “Test validity”	163
Cochrane Library	MeSH	MeSH “Exercise” OR MeSH “Sports”	MeSH “Sedentary Behavior”	MeSH “Child” OR MeSH “Adolescent”	MeSH “Schools”	MeSH “Surveys and Questionnaires”	MeSH “Reproducibility of Results”	779
