# Supplementary material for: Criterion and Convergent Validity of Youth Physical Activity and Sedentary Behavior Questionnaires in School Settings: A Systematic Review of Current Evidence and Future Perspectives
Source: Children (Basel). 2026 Jul 15;13(7):931. doi: 10.3390/children13070931 (PMC13406170; doi:10.3390/children13070931)
Supplement: Supplementary file 1 [file children-13-00931-s001.zip › Table S2 Quality assessment.pdf]

Table S2. COSMIN Study Design checklist for Patient-reported outcome measurement (PROM) instruments.

Study	Measurement Property									
	General recommendation	Content validity	Structural validity	Internal consistency	Reliability	Measurement error	Criterion validity	Hypotheses construct validity	Responsiveness	Translation process
Scholes et al.	Yellow	Black	Black	Black	Black	Black	Black	Green	Black	Black
Saint-Maurice et al. (2017)	Green	Black	Black	Black	Black	Black	Black	Yellow	Black	Black
McVeigh et al.	Orange	Black	Black	Black	Yellow	Black	Black	Orange	Black	Black
Jago et al.	Green	Black	Yellow	Green	Black	Black	Black	Green	Black	Black
Fillon et al.	Yellow	Black	Black	Orange	Orange	Orange	Black	Yellow	Black	Black
Foley et al.	Green	Black	Black	Black	Black	Black	Orange	Black	Black	Black
Bringolf-Isler et al.	Yellow	Black	Black	Black	Orange	Black	Black	Green	Black	Black

Study	Measurement Property									
	General recommendation	Content validity	Structural validity	Internal consistency	Reliability	Measurement error	Criterion validity	Hypotheses construct validity	Responsiveness	Translation process
Treuth et al. (2004)	green	dark grey	dark grey	dark grey	yellow	dark grey	dark grey	green	yellow	dark grey
Fairclough et al.	green	dark grey	dark grey	dark grey	dark grey	dark grey	dark grey	yellow	dark grey	dark grey
Vandoni et al.	yellow	dark grey	dark grey	dark grey	dark grey	dark grey	dark grey	yellow	dark grey	dark grey
Belton et Donncha	green	dark grey	dark grey	dark grey	orange	dark grey	dark grey	yellow	dark grey	dark grey
Ridley et al.	green	purple	dark grey	dark grey	orange	dark grey	dark grey	orange	dark grey	dark grey
Trost et al.	yellow	dark grey	dark grey	dark grey	dark grey	dark grey	dark grey	yellow	dark grey	dark grey
Saint-Maurice et al. (2015)	yellow	dark grey	dark grey	dark grey	dark grey	dark grey	dark grey	green	dark grey	dark grey

Study	Measurement Property									
	**General recommendation**	**Content validity**	**Structural validity**	**Internal consistency**	**Reliability**	**Measurement error**	**Criterion validity**	**Hypotheses construct validity**	**Responsiveness**	**Translation process**
Wang et al.	Very good	Not assessed	Not assessed	Not assessed	Adequate	Not assessed	Not assessed	Very good	Not assessed	Not assessed
Kastelic et al.	Very good	Not assessed	Not assessed	Not assessed	Adequate	Not assessed	Not assessed	Adequate	Not assessed	Not assessed
Treuth et al. (2003)	Very good	Not assessed	Not assessed	Not assessed	Adequate	Not assessed	Not assessed	Adequate	Not assessed	Not assessed
Rangul et al.	Very good	Not assessed	Not assessed	Not assessed	Adequate	Not assessed	Not assessed	Doubtful	Not assessed	Not assessed
Chinapaw et al.	Very good	Not assessed	Not assessed	Not assessed	Adequate	Not assessed	Not assessed	Adequate	Not assessed	Not assessed
Aggio et al.	Very good	Very good	Not assessed	Very good	Adequate	Not assessed	Not assessed	Adequate	Not assessed	Not assessed
Slinde et al.	Very good	Not assessed	Not assessed	Not assessed	Not assessed	Not assessed	Not assessed	Adequate	Not assessed	Not assessed
Arvidsson et al.	Very good	Not assessed	Not assessed	Not assessed	Not assessed	Not assessed	Not assessed	Doubtful	Not assessed	Not assessed

Four-point scale: Very good ●, Adequate ●, Doubtful ●, Inadequate ●, Not assessed ●
